# Marine-Derived Peptides from *Phaeodactylum tricornutum* as Potential SARS-CoV-2 Mpro Inhibitors: An *In Silico* Approach

**DOI:** 10.3390/microorganisms13061271

**Published:** 2025-05-30

**Authors:** David Mauricio Cañedo-Figueroa, Marco Antonio Valdez-Flores, Claudia Desireé Norzagaray-Valenzuela, Loranda Calderón-Zamora, Ángel Radamés Rábago-Monzón, Josué Camberos-Barraza, Alma Marlene Guadrón-Llanos, Alberto Kousuke De la Herrán-Arita, Verónica Judith Picos-Cárdenas, Alejandro Camacho-Zamora, Alejandra Romero-Utrilla, Carlos Daniel Cordero-Rivera, Rosa María del Ángel, Moisés León-Juárez, José Manuel Reyes-Ruiz, Carlos Noe Farfan-Morales, Luis Adrián De Jesús-González, Juan Fidel Osuna-Ramos

**Affiliations:** 1 Faculty of Medicine, Autonomous University of Sinaloa, Culiacán 80246, Mexico; davidmauricio013@gmail.com (D.M.C.-F.);; 2 Programa de Maestría en Ciencias en Biomedicina Molecular, Facultad de Medicina, Universidad Autónoma de Sinaloa (UAS), Culiacán 80246, Mexico; 3 Faculty of Biology, Autonomous University of Sinaloa, Culiacán 80019, Mexico; 4 Departamento de Anatomía Patológica, Instituto Mexicano del Seguro Social (IMSS), Culiacán 80200, Mexico; 5 Department of Infectomics and Molecular Pathogenesis, Center for Research and Advanced Studies (CINVESTAV-IPN), Mexico City 07360, Mexico; 6 Laboratorio de Virología Perinatal y Diseño Molecular de Antígenos y Biomarcadores, Departamento de Inmunobioquímica, Instituto Nacional de Perinatología, Ciudad de México 11000, Mexico; 7 Unidad Médica de Alta Especialidad, Hospital de Especialidades No. 14, Centro Médico Nacional “Adolfo Ruiz Cortines”, Instituto Mexicano del Seguro Social (IMSS), Veracruz 91897, Mexico; 8 Facultad de Medicina, Región Veracruz, Universidad Veracruzana, Veracruz 91700, Mexico; 9 Departamento de Ciencias Naturales, Universidad Autónoma Metropolitana (UAM), Unidad Cuajimalpa, Ciudad de México 05348, Mexico; 10 Unidad de Investigación Biomédica de Zacatecas, Instituto Mexicano del Seguro Social, Zacatecas 98000, Mexico

**Keywords:** antiviral peptides, SARS-CoV-2, main protease (Mpro), *Phaeodactylum tricornutum*, *in silico* analysis

## Abstract

The ongoing threat of viral pandemics such as COVID-19 highlights the urgent need for novel antiviral therapeutics targeting conserved viral proteins. In this study, peptides of 10–30 kDa derived from the marine diatom *Phaeodactylum tricornutum* were identified as potential inhibitors of SARS-CoV-2 main protease (Mpro), a key enzyme in viral replication. Peptides less than 60 amino acids in length were retrieved from the UniProt database and aligned with reference antiviral sequences using the Biopython pairwise2 algorithm. Six candidates were selected for structural modeling using AlphaFold2 and Swiss-Model, followed by molecular docking using ClusPro2. LigPlot+ was used to assess molecular interactions, while NetMHCpan 4.1 and AVPpred evaluated immunogenicity and antiviral potential, respectively. Molecular dynamics simulations over 100 ns were conducted using OpenMM. These peptides demonstrated stable binding interactions with key catalytic residues of Mpro. Specifically, peptide A0A8J9SA87 interacted with Cys145 and Glu166, while peptide A0A8J9SDW0 exhibited interactions with His41 and Phe140, both of which are known to be essential for Mpro inhibition. Although peptide A0A8J9X3P8 also interacted with catalytic residues, it exhibited greater structural fluctuations during molecular dynamics simulations and achieved lower AVPpred scores, suggesting lower overall antiviral potential. Therefore, A0A8J9SA87 and A0A8J9SDW0 were identified as the most promising candidates. Molecular dynamics simulations further supported the high structural stability of these peptide-Mpro complexes over a 100 ns timescale, reinforcing their potential as effective inhibitors. These findings support *P. tricornutum* as a valuable source of antiviral peptides and demonstrate the feasibility of *in silico* pipelines for identifying therapeutic candidates against SARS-CoV-2.

## 1. Introduction

Coronaviruses are RNA viruses that can infect a wide variety of hosts, from birds to mammals, allowing them to cause outbreaks at different times and regions of the world [1]. In 2002, the first case of severe acute respiratory syndrome (SARS) was reported in Foshan, China, which resulted in more than 8000 confirmed cases and 919 deaths in just one year. Subsequently, in 2012, Middle East respiratory syndrome (MERS) emerged, an infection with an even higher mortality rate, approximately 40% higher than that of SARS [2].

In 2019, a new variant of the SARS-CoV-2 virus caused a global outbreak of atypical pneumonia in Wuhan, China. Although it had a lower mortality rate than its predecessors, its high contagion and mutation capacity triggered a global pandemic that lasted until May 2023, when the World Health Organization (WHO) declared its official end [3].

The disease caused by SARS-CoV-2, known as COVID-19, presents with symptoms similar to those of seasonal influenza, including fever, cough, fatigue, and loss of taste or smell. By March 2024, the World Health Organization reported over 774 million confirmed cases and more than 7 million deaths, primarily affecting older adults and individuals with comorbidities such as cardiovascular disease, obesity, immunosuppression, and diabetes [4]. Additionally, post-infection complications, including neuropathies, have been increasingly recognized for their impact on patients’ quality of life [5].

Given the pandemic potential of coronaviruses, understanding their structural and molecular characteristics is critical. While vaccination has significantly reduced mortality, it has not substantially lowered transmission rates or addressed the virus’s high mutation frequency [6]. Thus, antiviral drug development should focus on compounds that target highly conserved viral proteins and exhibit properties, such as low toxicity, scalability, sustainability, and broad-spectrum efficacy [7,8].

Marine organisms, particularly microalgae, are a promising source of novel antiviral compounds. Notably, marine-derived cytarabine from sponges has proven effective in cancer therapy [9], and several microalgal species, including *Arthrospira*, *Chlorella*, *Dunaliella*, and *Haematococcus pluvialis,* have demonstrated antiviral activity against Mayaro virus [10]. In addition, *Spirulina*-derived phycobiliproteins have been investigated for their therapeutic potential [11].

Recent studies have also shown that protein hydrolysates of 10–30 kDa from the microalga *Phaeodactylum tricornutum* significantly reduced infection by dengue virus serotype 2 (DENV-2) [12], highlighting the potential of marine peptides in antiviral applications.

In this context, the present study proposes a full *in silico* approach to identify antiviral peptides derived from *Phaeodactylum tricornutum* with potential activity against the main protease (Mpro) of SARS-CoV-2, which is a key enzyme in the viral replication process. Using a combination of bioinformatic tools, including sequence alignment, structural modeling, molecular docking, molecular dynamics, immunogenicity prediction, and antiviral activity screening, we aimed to identify candidate peptides for future in vitro validation and therapeutic development.

## 2. Materials and Methods

### 2.1. Retrieving and Aligning Peptide Sequences

Peptide sequences from *Phaeodactylum tricornutum* were obtained from the UniProt database (www.uniprot.org), accessed on 15 May 2024. The dataset was obtained and processed using a data management library to guarantee appropriate formatting. Each peptide sequence was aligned with the dataset, and the alignment scores ranked the outcomes. The five highest-scoring alignments for each peptide were chosen, along with information such as organism, sequence length, molecular weight, and protein name. Peptide sequences containing less than 60 amino acids and with molecular weights below 30 kDa were selected because these criteria characterize conventional short peptides of significance [13]. Alignment was performed on Google Colab using Python 3.10 and the Biopython library 1.83, employing the pairwise2.align.globalxx function with reference sequences as templates (Table 1).

This approach ensures that the entire sequence length is considered during alignment. Each peptide was aligned against the complete *P. tricornutum* proteome to examine potential matches. Alignment scores ranked matches, providing quantitative similarity measures. The five highest-scoring alignments per peptide were retained.

### 2.2. Peptidic Modeling

Two computational approaches were applied to model peptide structures using AlphaFold2 (https://www.deepmind.com/alphafold) accessed on 1 June 2024 and Swiss-Model (https://swissmodel.expasy.org) accessed on 1 June 2024. AlphaFold2 employs a neural network-based method to generate structural models, leveraging multiple sequence alignments and deep learning to predict highly accurate protein structures. The reliability of these models was assessed based on the Interface Predicted Template Modeling (ipTM) score, considering values above 0.8 as reliable indicators of structural accuracy [14].

Swiss-Model, in contrast, utilizes homology-based modeling, in which template identification is performed through sequence similarity searches in the PDB database. Global Model Quality Estimate (GMQE) and QMEANDisCo scores were calculated to evaluate model reliability, incorporating statistical potential and experimental data. The highest-scoring models, based on the QMEAN and GMQE assessments, were selected for the subsequent docking analysis. These complementary modeling approaches ensure structural accuracy, providing a robust foundation for downstream molecular docking and dynamic simulations [15,16].

### 2.3. Molecular Docking

Molecular docking was performed using the ClusPro2 server hosted by Boston University. The ClusPro2 server was specifically designed for peptide–protein docking. For this experimental strategy, because the peptide chain identifiers were previously modified using PyMol 2.6, the server was configured to run on a CPU with no chain restrictions. The receptor was identified using the PDB code (ID: 6LU7).

### 2.4. Residue Interaction Analysis

Local software was used to examine the interactions of the top ten models obtained from the ClusPro2 docking analysis. Specifically, LigPlot+ version 2.2.8 was used to delineate these interactions. LigPlot+ uses the Dimplot function, which was designed for peptide–receptor binding analysis. LigPlot+ provides a schematic representation of the interactions between amino acid residues in two dimensions and categorizes them based on hydrophobic contacts, hydrogen bonds, and binding distances [17].

### 2.5. Immunogenicity

Servers capable of predicting major histocompatibility complex (MHC) interactions were used to assess whether these peptides could induce an immune response, specifically the NetMHCpan-4.1 server. This server uses over 850,000 reported peptide sequences and their binding affinities, as well as experimental data from mass spectrometry-eluted ligands to make predictions based on an artificial neural network (ANN) algorithm [18].

### 2.6. Molecular Dynamics

Molecular dynamics (MD) simulations were performed to assess the stability and dynamic behavior of peptide–Mpro complexes over a 100 ns timespan. Simulations were conducted using OpenMM with the AMBERff19SB force field, employing high-performance cloud computing resources via Google Colab, in accordance with the computational framework outlined in “Making it Rain: Cloud-Based Molecular Simulations for Everyone” [19].

The MD simulations were conducted using the script created by Arantes, which is accessible in the public GitHub notebook. The simulation framework was designed to operate in Google Colab, allowing for the efficient execution of 100 ns molecular dynamics using the AMBER force field [19]. The ff19SB force field, an improved model optimized for protein–peptide dynamics, was employed because of its superior performance in refining backbone conformations and resolving solvation inconsistencies compared with earlier models, such as ff99SB and ff14SB [20]. The OPC water model was selected for solvation because it offers enhanced accuracy, reduced error compensation, and improved behavior of intrinsically disordered proteins [21].

The preparation of the system included energy minimization and equilibration steps, ensuring proper solvation and temperature–pressure stabilization. The LEaP module from AMBER was used for system parameterization, in which explicit solvation with an OPC waterbox and periodic boundary conditions were applied. Electrostatic interactions were handled using the Particle Mesh Ewald (PME) method, and bond constraints were imposed on the hydrogen atoms. Equilibration was performed in an NPT ensemble, where the temperature was maintained at 298 K using a Langevin thermostat and the pressure was controlled using a Monte Carlo barostat.

The production phase of the simulation was executed using a 2 fs time step, with trajectories recorded at 10 ps intervals. Root mean square deviation (RMSD) and root mean square fluctuation (RMSF) analyses were performed using MDAnalysis and PyTraj to assess the conformational stability of peptide-bound and unbound Mpro structures. The resulting trajectories were processed and concatenated using MDAnalysis, and the protein–ligand interaction dynamics were visualized using Py3Dmol.

### 2.7. Use of Antiviral Peptide Prediction Server (AVPpred)

The antiviral potential of the selected peptides was evaluated using the AVPpred server, a web-based tool designed to predict antiviral peptides. AVPpred utilizes a support vector machine (SVM) algorithm trained on a dataset of 1245 experimentally validated antiviral peptides, achieving a Matthew’s Correlation Coefficient (MCC) of 0.70 [22]. The prediction process involves sequence alignment, composition-based models, physicochemical properties, and classification of peptides that surpass a predefined physicochemical threshold as antiviral candidates.

AVPpred was used to analyze three specific peptide sequences: (A) A0A8J9SA87, (B) A0A172E6W5, and (C) TVNVLAWLY. Peptides A and B, previously identified through molecular docking, were evaluated for their potential antiviral activity, whereas peptide C was analyzed because of its reported epitope interaction with SARS-CoV-2 [23]. The predictive performance of AVPpred was validated using a 5-fold cross-validation technique to ensure robustness in antiviral classification [22]. Hydrophobicity and hydrophilicity ratios were calculated to assess the membrane interaction potential and prioritize peptides with high AVP scores for further validation.

**Table 1:** Results of the peptidic alignment performed in Google Colab.

Peptide Reference	UniprotID	Score	Name	Peptide Alignment
QTFSVLACY [23]	A0A8J9SAR9	9	Unknown	NESEQGRIQGALFSLQALASATGPMLLRFIYHLTKDG AFLGPGSMFVVASGIYLIAVYCAYSLP
QTFSVLACY [23]	A0A8J9TDK2	9	Unknown	QIGTVVKANYCLWPFFQYINFTFVPSSLRV LATNLMSVLWNCYFCSCIA
QTFSVLACY [23]	A0A172E6W5	8	H(+) export diphosphatase	VRGAPDSELQGKGSDIHKAAVVGDTVGDPFKD TSGPALNIVMKLMAVLSLVFADTFAVNNGQGLLNLA
TVNVLAWLY [23]	A0A8J9SDW0	9	Unknown	MTVSNEESPDVIELDASTTVETIKIAPTEWIKRLQSTW GEPLVVPEWEDDTEGYRAKNGWQA
TVNVLAWLY [23]	A0A8J9SA87	8	G-domain protein	MKLQTAIVGLPNVGKSTLFNALTETQGAEAANYPFCTIEPN
TVNVLAWLY [23]	A0A8J9X3P8	8	Protein with protein kinase domain	NPKRTTKVNLGRVLKTLVHVHGLQLMQDGVFNAD PHPGNVLVLPDGRLGLLDYGMV
YLQYAVLRHKRREC [24]	A0A8J9SA66	11	Protein with protein kinase domain	VYL-AADVMLPLLQRMHEAGVVHRDVKPSNCV RSTGERDFCIVDFGLSK
YLRYKCLCTWQITVC [24]	A0A8J9X2W5	11	Unknown	MQSGAMYEFLFSYKSTQTLPGIPTSGVPRNWRGPLGAQE WAVRRTDRNQWEDGLVFLCTS
YLRYKCLCTWQITVC [24]	A0A8J9X430	11	Impact N-terminal domain-containing protein	FAYRLTETISDGTRVSKHDNDDDGEYGAGSKLAHLLQ VRDEKDVVVLVARWFGGVHL
YSVAQKRKYWLFVLC [24]	A0A8J9SNS2	11	Bromine domain protein	FLNPVTDEIAPGYSKVIKHPICIA-AMEDK-VESHKYNSPSDW —EGDVNLMYKNCIDYNRGN
YSWTYLGRDYYWSC [24]	A0A8J9TRC1	10	Protein with Arf-GAP domain	MAIYEKELNTAS-NTVYEMKTADYAELLSMPG-NSVCAD —CGAVNPNWGSPKLGILFCTDCSGKH
YSWTYLGRDYYWSC [24]	A0A8J9T715	10	N-terminal SNF2 protein	AKEAWLEFRDKLYDPNEPHS—Y-KNGNRLRD -YQVEGVNWLASTWYKKQGCILADEMGLGK
YTKHVYYHITYILYVC [24]	A0A8J9T326	11	USP domain protein	DGHY-KCHV-QHQATRQWYEIQDL- HVQEIMPQQIGLSECYLLIFRKSGL
YVHPKLHKCCIYIVWC [24]	A0A8J9TWV5	10	Protein with protein kinase domain	VYLAADV-MLP-LLQRMHEAGVVHRDVKPSNCVRSTGERDFC —IV—DFGLSK

## 3. Results

### 3.1. Alignment

The peptide sequences of the diatom *P. tricornutum* were retrieved from the UniProt database (www.uniprot.org) accessed on 15 May 2024. Only sequences with less than 60 residues were included in the analysis. Table 1 presents the alignment results for the 16 peptides using the pairwise2.align.globalxx method.

In Table 1, the “peptide reference” column indicates the template peptide used for alignment. The ‘UniProt ID’, ‘Name’ and ‘Molecular Weight’ columns contain data obtained from the UniProt database (www.uniprot.org/uniprotkb). The “Score” and “Peptide alignment” columns display the results generated by the code executed on Google Colab. Peptides with scores less than or equal to 9 were selected for further evaluation. Scores of 10 or higher indicated that gaps were introduced between alignment matches, suggesting that these peptides exhibited neither omissions nor mismatches. Therefore, peptides with the following UniProt IDs—A0A8J9SAR9, A0A8J9TDK2, A0A172E6W5, A0A8J9SDW0, A0A8J9SA87, and A0A8J9X3P8—were chosen for subsequent analyses.

### 3.2. Peptidic Modeling

The peptide sequences were modeled using two methods: AlphaFold2 (alphafoldserver.com) and Swiss-Model (swissmodel.expasy.org). The former relies on an ANN algorithm [25], whereas the latter is based on sequence homology. The results obtained from AlphaFold modeling are summarized in Table 2, whereas those obtained from homology modeling are presented in Table 3.

The quality of AlphaFold peptide modeling was primarily assessed using the ipTM metric, except A0A8J9X3P8, which scored below 0.7 on this scale [15].

The DeppMind team recommends an optimal ipTM value above 0.8 to consider a model reliable. Similarly, Scardino et al. used AlphaFold models for molecular docking and concluded that these models were not well suited for this type of analysis [16].

In homology modeling, two key variables must be considered to determine the reliability of the models. The first is the GMQE (Global Model Quality Estimate) score, which is based on a multilayer perception approach. Additionally, the IDDT score of the resulting model was calculated by combining the structural quality and template quality [13].

**Figure 1:**
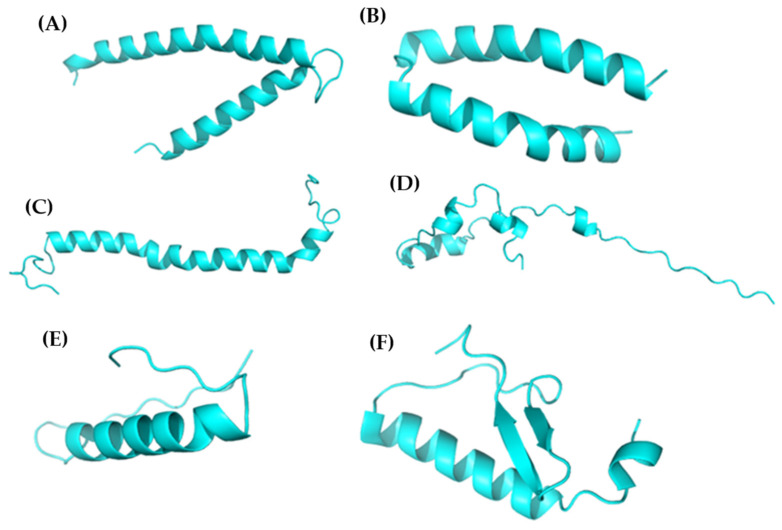
Models of six peptides with low alignment scores: (**A**) A0A8J9SAR9, (**B**) A0A8J9TDK2, (**C**) A0A172E6W5, (**D**) A0A8J9SDW0, (**E**) A0A8J9SA87, and (**F**) A0A8J9X3P8.

The IDDT also represents the root square difference between the IDDT score and pairwise residual–residue distance estimate of the high-scoring homology structure, with prediction fidelity related to the model size [15]. Prediction error estimates were obtained using models similar to the input sequence, which did not incorporate the AlphaFold data. The models obtained are shown in Figure 1.

### 3.3. Molecular Docking

Molecular docking analyses were performed on the Boston University ClusPro2 server (www.cluspro.bu.edu) accessed in 4 August 2024 [26] using the SARS-CoV-2 main protease (Mpro, PDB: 6LU7) as the target protein and Swiss-Model. The results of these analyses are presented in Table 4.

The table shows the peptides with the lowest cluster memberships (A0A8J9X3P8 and A0A172E6W5) and lowest weighted scores (A0A8J9X3P8, A0A172E6W5, and A0A8J9SA87), which were selected for subsequent analyses. The results indicated that peptides A0A8J9SAR9, A0A8J9TDK2, and A0A8J9SA87 had the highest number of cluster members, suggesting a more stable docking. This indicates that the interaction between the peptide and ligand was highly spontaneous. However, for classification purposes, the authors suggested that the most feasible approach is to select the peptide with the largest number of clusters [27].

**Figure 2:**
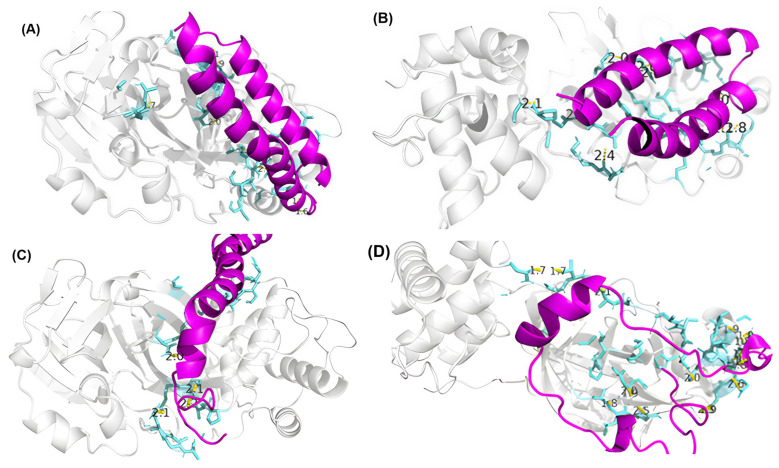

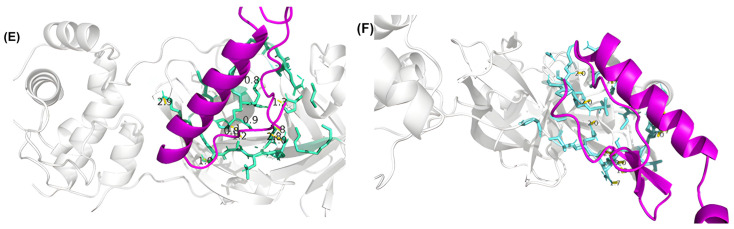
Molecular visualization of peptides and Mpro in PyMol: (**A**) A0A8J9SAR9, (**B**) A0A8J9TDK2, (**C**) A0A172E6W5, (**D**) A0A8J9SDW0, (**E**) A0A8J9SA87, and (**F**) A0A8J9X3P8.

PyMol molecular visualization software was used to provide an initial overview of the peptide–protein interactions, complementing the ClusPro results. Figure 2 illustrates these interactions. The modeled peptides are in magenta, the white region is the complete Mpro structure, and the blue region represents the residues that interact with the peptides.

### 3.4. Residue Interactions

Based on the work of Jin et al. [28], three-dimensional models of Mpro (PDB: GLU7) and its inorganic inhibitor N3 were downloaded from the Protein Data Bank (www.rcsb.org) accessed in 29 July 2024. Using PyMOL, the amino acid residues that interfered with the N3 inhibitor were identified: T24, T25, T26, M41, M49, F140, L141, N142, G143, S144, C145, H163, H164, M165, E166, P168, H172, D187, Q189, T190, A191, and Q192. In particular, the key roles of C145, H41, G143, S144, and E166 reported in [29] have been emphasized.

**Figure 3:**
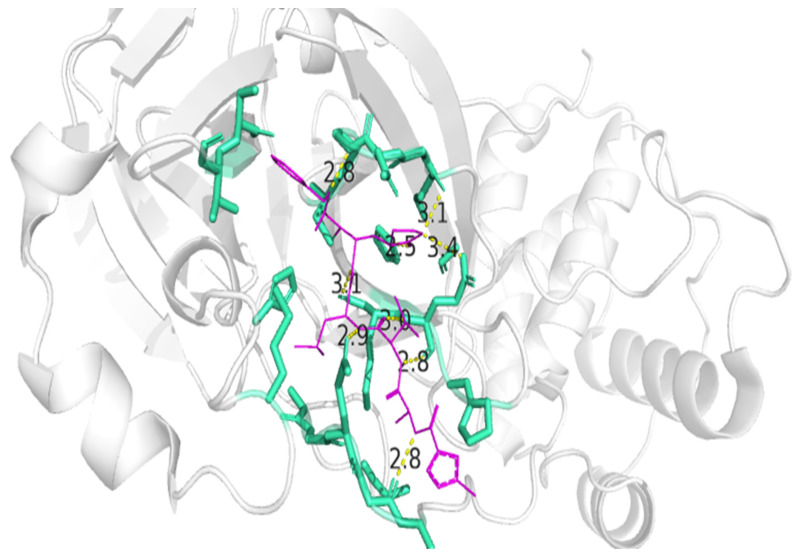
Interactions between N3 and the Mpro active site as visualized in PyMol.

The PyMol model is shown in Figure 3, in which the residues that interact with N3 are highlighted, in aqua green color, residues associated with catalysis are identified as follows.

**Figure 4:**
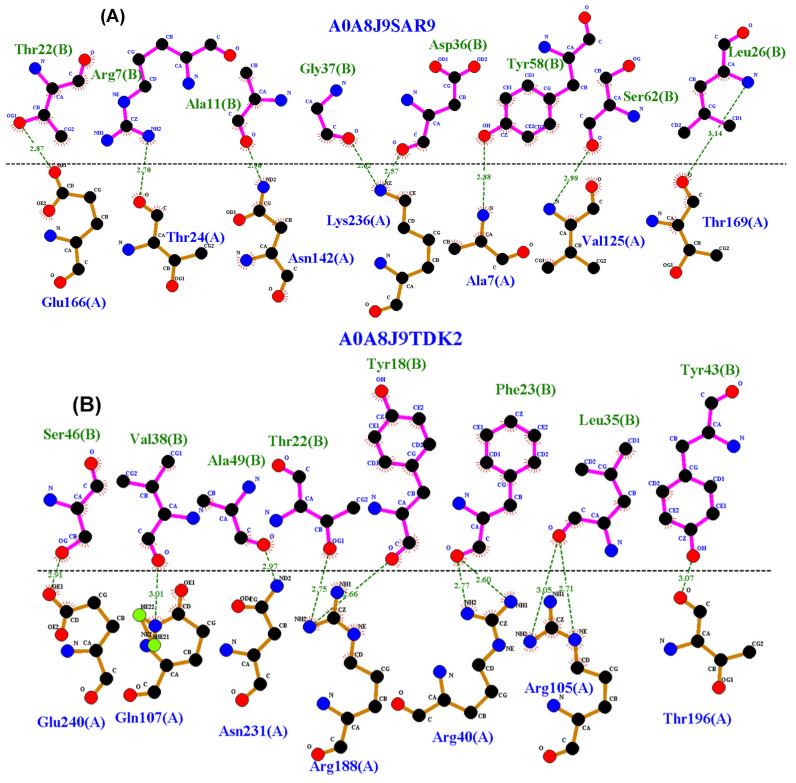

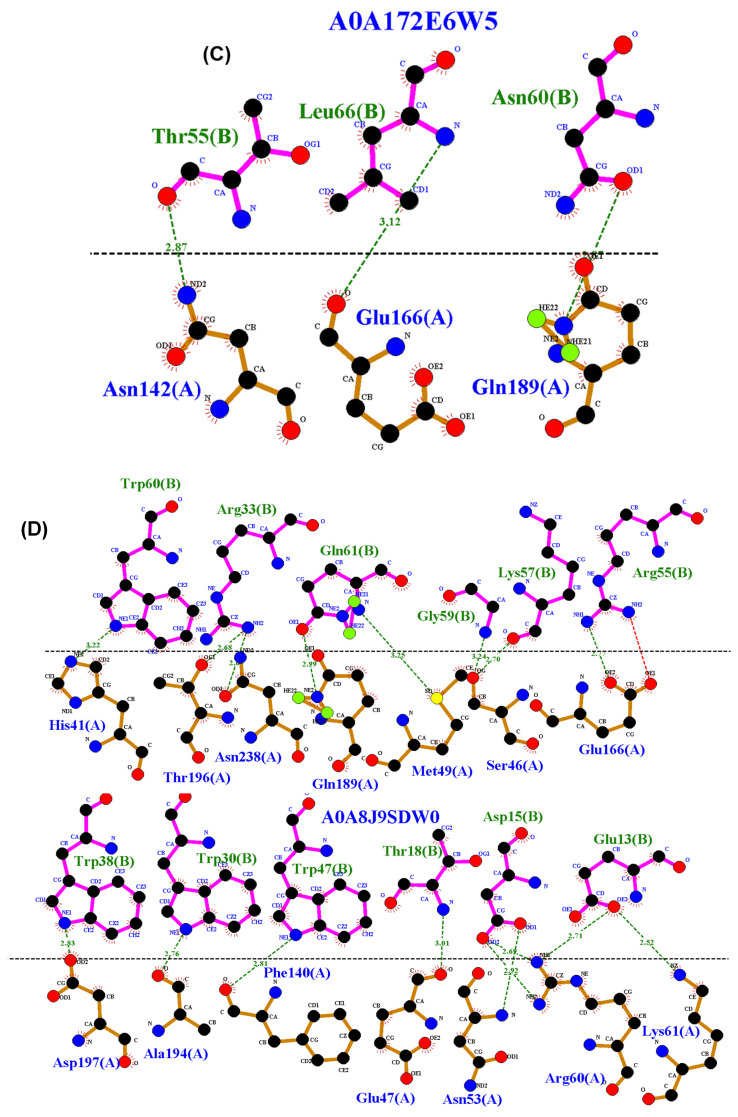

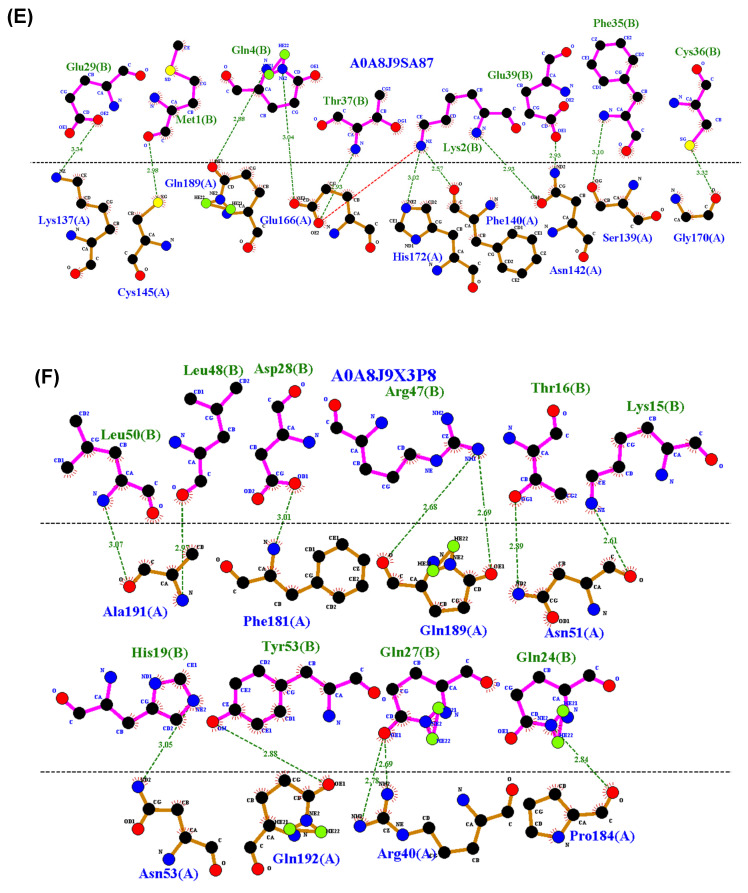
Visualization with LigPlot+ 2.2 of peptides obtained from molecular docking interacting with Mpro: (**A**) peptide A0A8J9SAR9 interacts with Glu166 and Asn142 (active and stabilizer sites), (**B**) peptide A0A8J9TDK2 did not show significant interactions, (**C**) peptide A0A172E6W5 residues interact with Glu166, Gln189, and Asn142, all of which are involved in Mpro catalysis, (**D**) peptide A0A8J9SDW0 residues interact with His41, Phe140, and Met49, with the first two considered key residues in N3 inhibition, (**E**) peptide A0A8J9SA87 interacts with Cys145 and Gln166, defined as key residues in the active site, in addition to Phe140, Asn142, and Gln189, which stabilize catalysis, and (**F**) peptide A0A8J9X3P8 residues interact with Gln192, Gln189, and Ala191, all of which are related to catalytic stabilization.

Using the model obtained from the molecular docking results and the “dim plot” option of LigPlot+ version 2.2.8, the interactions between Mpro and the investigated peptides were identified. The results are shown in Figure 4.

### 3.5. Immunogenicity

**Figure 5:**
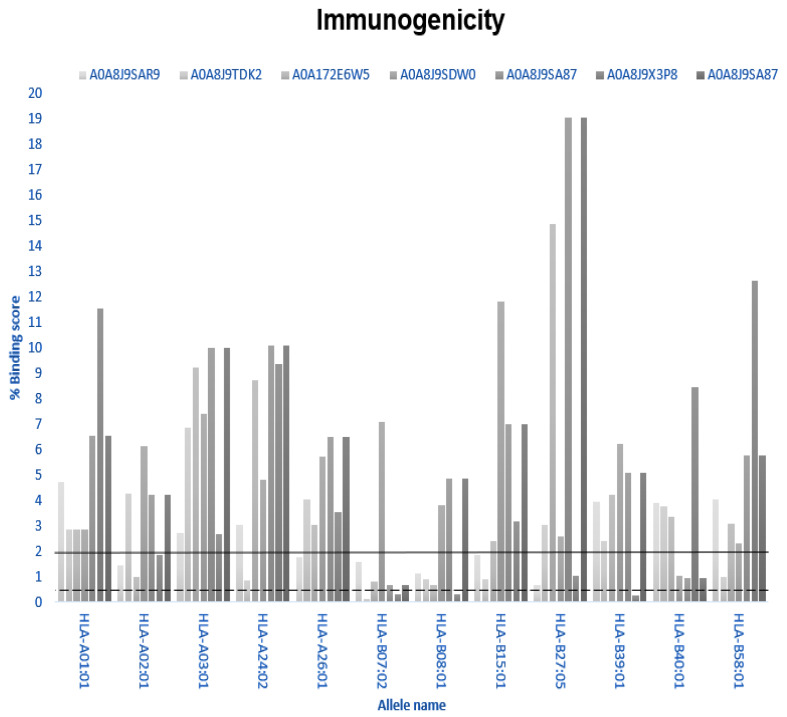
NetMHCpan 4.1 evaluation of six peptides across twelve human MHC alleles.

The results obtained from the NetMHCpan 4.1 server analysis are presented in Figure 4. These results illustrate how the evaluated peptides interact with various alleles of the major histocompatibility complex (MHC). According to NetMHCpan 4.1, interactions with binding values below 0.5% were classified as strong, represented by dotted line in Figure 5, whereas those with binding values of >0.5% and <2% were considered weak, represented by a solid line. Based on the interpretation shown in Figure 5, the weak interactions between peptide IDs and their corresponding allele names are summarized in Table 5.

Furthermore, these two peptides exhibited strong interactions. The first peptide (ID: A0A8J9TDK2) interacted with the HLA-B07:02 allele, whereas the second peptide (ID: A0A8J9X3P8) interacted with HLA-B07:02, HLA-B08:01, and HLA-B39:01. According to the literature, these storage interactions can potentially elicit an immune response.

The simulation demonstrates how Mpro behaves under different conditions over 100 ns, using the Mpro and Mpro + N3 models as comparative references (green and magenta, respectively). Notably, the magenta trace (Mpro + N3) shows that after 60 ns, the complex experienced an approximate increase of 2 Å RMSD, suggesting structural modification; however, it remained stable thereafter.

**Figure 6:**
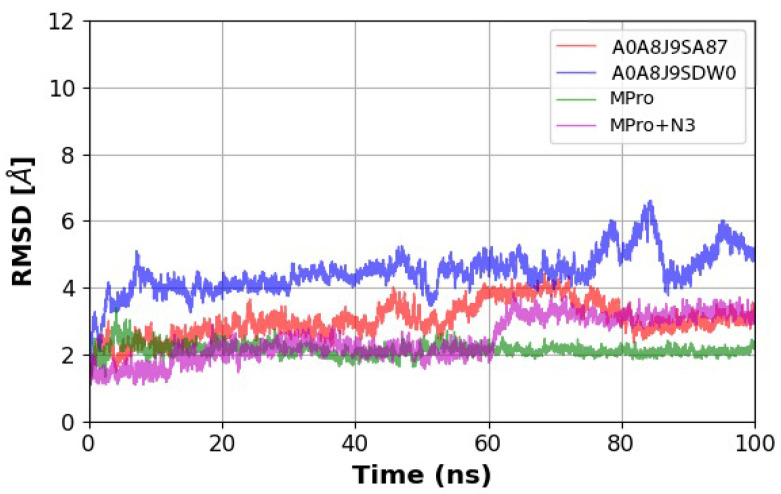
Results of a 100 ns molecular dynamics simulation, where the root mean square deviation (RMSD) values were obtained for four different compounds: peptide A0A8J9SA87 (red), peptide A0A8J9SDW0 (blue), Mpro (green), and Mpro + N3 (magenta), and the RMSD of the peptides A0A8J9SA87 (red), A0A8J9SDW0 (blue), Mpro (green), and Mpro + N3 complex (magenta).

Furthermore, the peptides A0A8J9SA87 (red) and A0A8J9SDW0 (blue) exhibited consistent stability. In particular, the red trace presents a low and steady RMSD distribution throughout the simulation, whereas the blue trace reveals a transient increase of approximately 3 Å around 75 ns, lasting approximately 10 ns, which may indicate a potential tendency toward further structural changes (Figure 6).

### 3.6. Use of Antiviral Peptide Predicton Server (AVPpred)

The results for the peptides evaluated were intriguing. Peptide A0A8J9SDW0 exhibited over 64% antiviral prediction based on its physicochemical properties, whereas its composition model yielded only approximately 19%, and no alignment with known antiviral peptides was detected; therefore, it was classified as a non-AVP in the alignment analysis. Consequently, this peptide received an overall AVP prediction score of 1, as only physicochemical analysis surpassed the threshold.

In contrast, peptide A0A8J9SA87 showed similar results. In the physicochemical analysis, despite its notably shorter sequence, it achieved a prediction percentage exceeding 63%, surpassing the threshold. However, both alignment and composition analyses indicated low efficiency, being classified as a non-AVP in the alignment model and yielding a composition value below 22%, resulting in an overall AVP prediction score of 1.

Moreover, as reported by Marriam et al., the reference peptide used for nesting did not exceed the threshold in any prediction model, with the highest value obtained in the composition model, which remained below 50% [23].

Additionally, none of the three peptides exhibited structural motifs that significantly reduced the predicted AVP values. Regarding polarity, both peptides A0A8J9SDW0 and A0A8J9SA87 displayed similar hydrophobic and hydrophilic profiles, with a nearly 50/50 ratio, which was expected given their closely matched physicochemical percentages. The results are summarized in Table 6.

## 4. Discussion

The global search for effective antiviral therapies is a priority in biomedical research. The COVID-19 pandemic, caused by SARS-CoV-2, has underscored the urgent need for innovative and accelerated drug discovery methods. Traditional drug development is a time-consuming and expensive process that requires molecular identification for regulatory approval. During the pandemic, innovative technologies, including machine learning and artificial intelligence (AI), have been used to expedite this process by modeling molecular activity using *in silico* platforms. Notably, Guedes et al. (2024) introduced DockThor-VS, a web server designed for the virtual screening of receptor–ligand interactions, demonstrating the growing importance of computational methods in early-stage drug discovery [30].

In this study, we implemented a fully virtual (*in silico*) approach to explore the antiviral potential of peptides derived from the marine diatom *Phaeodactylum tricornutum*. Our objective was to identify peptides with a high affinity for the main protease (Mpro) of SARS-CoV-2, a key enzyme in viral replication. To achieve this, we combined several bioinformatics tools, including sequence alignment, structural modeling, molecular docking, molecular dynamics (MD) simulations, immunogenicity assessment, and antiviral activity prediction. Each step was designed to minimize methodological bias and leverage the computational power of cloud-based platforms such as Google Colab.

Our investigation began by aligning *P. tricornutum* derivate peptides (≤60 amino acids) utilizing the pairwise2.align.globalxx function within the library created for reference. Sixteen peptides were initially selected based on their alignment scores, culminating in a final selection of six peptides with alignment scores of ≤9, signifying minimal deletions or mismatches. Reduced alignment scores signified fewer discrepancies, indicating greater similarity between peptides and aligned proteins. This system detects closely related sequences within the *P. tricornutum* proteome and offers insights into evolutionary connections and functional resemblances. This alignment method was previously validated in the creation of AVP-GPT, a transformer-based AI system for designing antiviral peptides that exhibited enhanced performance relative to models such as LSTM and SVM in terms of perplexity and AUC values [31]. The integration of sequence alignment, scoring, and metadata collection establishes a framework for analyzing peptides from *P. tricornutum*, facilitating insights into the protein landscape of the organism.

Structural modeling was subsequently performed using two distinct methodologies: AlphaFold2 and Swiss-Model. Although AlphaFold2 provides initial visualizations of peptide structures, its models were excluded from further analyses because of low ipTM scores (<0.8), as recommended by DeepMind [25]. Moreover, it has been established that models generated using AlphaFold exhibited diminished reliability for molecular docking, thereby corroborating our choice to utilize Swiss-Model instead [16]. All selected peptides attained GMQE scores exceeding 0.6 according to the Swiss-Model, thereby qualifying them as structurally viable for molecular docking.

Docking analyses were performed using OpenMMserver with SARS-CoV-2 Mpro protein (PDB ID: 6LU7) as the target. The findings indicated that three peptides, A0A8J9SAR9, A0A8J9TDK2, and A0A8J9SA87, exhibited the greatest cluster membership, whereas peptides A0A8J9X3P8, A0A172E6W5, and A0A8J9SA87 had the most advantageous weighted energy scores. Prior research has shown that clustering and binding energy serve as accurate measures of interaction spontaneity and docking stability [27]. PyMOL visualization verified that the highest-performing peptides were situated around the active site of Mpro.

To further evaluate the binding interactions, we used LigPlot+ with the DimPlot function to analyze amino acid contact within the peptide–Mpro complexes. The peptides A0A8J9SDW0 and A0A8J9SA87 displayed multiple interactions with catalytically significant residues, such as Cys145, His41, Glu166, and Phe140, which have previously been shown to play critical roles in Mpro inhibition [28,29]. These observations suggest a strong potential for inhibitory activity, warranting further exploration of these candidates.

Molecular dynamics (MD) simulations were conducted for peptides A0A8J9SDW0 and A0A8J9SA87 complexed with Mpro. Simulations spanned 100 ns using OpenMM with the ff19SB force field and OPC water model, both of which demonstrated improved accuracy in modeling intrinsically disordered proteins [20,21]. RMSD analyses indicated that both peptide complexes maintained stable interactions with Mpro throughout the simulation, with RMSD values below 7 Å for A0A8J9SDW0 and below 5 Å for A0A8J9SA87. These stability metrics were consistent with those observed in MD simulations of known inhibitors [19].

To assess immunogenicity, NetMHCpan 4.1 was employed to evaluate peptide interactions with 12 human MHC alleles. Two peptides, A0A8J9TDK2 and A0A8J9X3P8, demonstrated weak interactions with multiple MHC class I alleles, and A0A8J9X3P8 exhibited strong interactions with HLA-B07:02, HLA-B08:01, and HLA-B39:01. These results indicate the potential for T-cell immune responses, which is a critical consideration for future vaccine-related applications [18,32,33].

Finally, antiviral potential was predicted using AVPpred, a tool that integrates alignment, composition, and physicochemical parameters. The peptides A0A8J9SDW0 and A0A8J9SA87 exceeded the threshold for physicochemical antiviral prediction (>63%) but were not classified as AVPs in alignment- or composition-based models, resulting in an overall score of 1. In contrast, the reference peptide used by others teams failed to meet the threshold for any category, reinforcing the potential of the selected candidates [23].

The results of this study demonstrated that peptides derived from *Phaeodactylum tricornutum* are potential inhibitors of the SARS-CoV-2 main protease (Mpro). Using a comprehensive *in silico* approach, we identified two promising peptides, A0A8J9SA87 and A0A8J9SDW0, which exhibited stable binding interactions with the critical catalytic residues of Mpro, such as Cys145, His41, and Glu166. Our findings corroborate the potential of marine peptides as antiviral agents, particularly against SARS-CoV-2, which is consistent with several recent studies.

For instance, similar research was conducted into marine algal antagonists targeting both the 3CL protease and the spike glycoprotein of SARS-CoV-2 using molecular dynamics simulations. Their research highlighted the potential of marine algal peptides to target viral proteases, offering a comparison with our own findings of stable peptide–Mpro interactions [34]. Similarly, metabolites from marine algae have been explored as high-affinity inhibitors of SARS-CoV-2 Mpro, using docking and molecular dynamics simulations. They found similar peptide interactions, confirming the suitability of Mpro as a drug target and supporting the findings of this study that peptides from marine organisms can effectively inhibit SARS-CoV-2 replication [35].

Other studies have employed similar methodologies to identify antiviral peptides that target viral proteases, including those of SARS-CoV-2. Reporting cyclic antiviral peptides that bind to the main protease of SARS-CoV-2, demonstrating the viability of targeting this protein for drug development [24]. Similarly, peptides from natural sources that exhibited potential against viral proteases, highlighting the vast potential of marine and other natural peptides as antiviral agents [34].

Our results also align with others where microalgal peptides were investigated microalgal peptides for their potential to target the spike protein of COVID-19. While their focus was on the spike protein, the computational methods they applied, including molecular docking, were highly comparable to those we used to target Mpro [34]. This cross-study approach enhances the credibility of our *in silico* findings and supports the broader scope of marine-derived peptides for targeting different components of the SARS-CoV-2 virus.

Moreover, the potential use of microalgal peptides extends beyond direct viral inhibition. The role of diatoxanthin compounds from marine microalgae has been explored, showing their impact on cytokine storms in SARS-CoV-2 infection models. This further highlights the potential of marine compounds in managing immune responses in addition to their antiviral activity. Their study supported the idea that marine peptides, including those investigated in our study, could also play a role in modulating immune responses in COVID-19 patients [36]. The dimensions of the chosen peptides aligned with the ranges documented for antiviral peptides *in silico* and the evidence presented by our team, specifically the in vitro studies on viral infections induced by dengue serotype 2 conducted by Rivera-Serrano et al., constitutes a critical factor in delineating the spectrum of peptides to be assessed *in silico*. [12].

Although our study supports the potential of *P. tricornutum*-derived peptides as SARS-CoV-2 Mpro inhibitors, there are several important limitations that should be addressed in future research. Notably, computational models, while robust, may still present discrepancies with actual biological behavior, as the inherent flexibility of proteins and their interactions with ligands cannot always be fully captured *in silico*. Additionally, the use of AlphaFold2 models, which demonstrated relatively low reliability for docking analyses in this study, emphasizes the need for high-quality, experimentally verified models, as also discussed by other authors, who concluded that AlphaFold models were less reliable for molecular docking applications because of their limited flexibility [16]. Thus, combining structural data from multiple sources, including homology modeling and molecular dynamics simulations, is critical to ensure a more accurate prediction of peptide–protein interactions.

The molecular dynamics simulations conducted in this study revealed the high stability of the peptide–Mpro complexes, with peptides A0A8J9SDW0 and A0A8J9SA87 showing minimal fluctuations in RMSD over a 100 ns timespan. These results are consistent with those of other teams, who observed similar dynamics for marine algae-derived inhibitors, confirming the long-term stability of peptide–protein complexes. This stability is crucial for the potential therapeutic development of these peptides as it ensures their viability as inhibitors over extended periods [37].

In addition, immunogenicity prediction via NetMHCpan 4.1 demonstrated that some of the peptides in our study, such as A0A8J9X3P8, have the potential to induce immune responses through interactions with various MHC alleles. These findings align with those reported in 2021, who found that microalgal peptides targeting the spike protein also exhibited immunogenic potential, suggesting their use in vaccine-related applications. The strong immunogenicity observed in some of our peptides further supports their potential use in therapeutic and vaccine contexts [38].

However, it is important to note that while these peptides show promise, AVPpred analysis classified them as non-AVPs based on alignment and composition-based models, which calls for additional validation. The prediction methods used, including physicochemical property-based scoring, suggested that these peptides could still have biological activity despite their lower classification scores. Similar limitations have been noted in other studies, where predictions based on peptide composition and alignment sometimes fail to capture the full antiviral potential of peptides [38,39].

Our results demonstrated the utility of combining multiple computational tools to identify promising antiviral peptide candidates. The workflow we present is cost-effective, reproducible, scalable, and suitable for rapid screening of large peptide libraries. The use of cloud-based platforms, such as Google Colab, has proven effective for both structural modeling and MD simulations [19,33], reducing the need for a high-performance local computing infrastructure.

Furthermore, this methodology aligns with recent trends in structure-based drug design, where fragment-based molecular modeling, docking, and analog generation are employed to develop novel inhibitors, as demonstrated in HIV-1 protease studies [40]. Similar approaches were used to optimize the peptide scaffolds identified in the present study.

However, despite these promising results, further validation is required. In vitro and in vivo studies are necessary to assess the antiviral efficacy and toxicity of these peptides. Specifically, evaluating various SARS-CoV-2 variants and investigating the potential synergistic effects of these peptides in conjunction with existing antiviral therapies could provide essential insights into therapeutic applications. Optimization of peptides to improve stability, bioavailability, and specificity is necessary for their potential transition into clinical development. Finally, the integration of *in silico* methods with high-throughput screening in experimental settings may lead to the identification of novel antiviral agents from marine sources. Future studies should explore the peptide potential of other microalgae and marine organisms, expanding the range of compounds available for combating viral infections.

## 5. Conclusions

This study presents a comprehensive *in silico* approach for the identification and characterization of antiviral peptides derived from the marine diatom *Phaeodactylum tricornutum* that target the SARS-CoV-2 main protease (Mpro).

By integrating sequence alignment, structural modeling, molecular docking, interaction analysis, molecular dynamics simulations, immunogenicity prediction, and antiviral activity screening, we identified two promising candidate peptides, A0A8J9SA87 and A0A8J9SDW0.

These peptides demonstrated stable binding interactions with key catalytic residues of Mpro. Specifically, peptide A0A8J9SA87 interacted with Cys145 and Glu166, while peptide A0A8J9SDW0 exhibited interactions with His41 and Phe140, both of which have been reported as critical residues for the inhibition of Mpro by the reference inhibitor N3. These interactions were confirmed through molecular docking and LigPlot+ visualization. Moreover, molecular dynamics simulations revealed high structural stability of the peptide–Mpro complexes over a 100 ns timescale, supporting their potential as effective inhibitors.

Although AVPpred analysis classified both peptides as non-AVPs in the alignment- and composition-based models, their high physicochemical antiviral scores suggest a strong likelihood of biological activity. Additionally, MHC binding predictions indicated weak-to-strong immunogenic responses for the selected peptides, which is essential for evaluating their suitability for therapeutic or vaccine-related applications.

Taken together, our findings support the potential of *P. tricornutum* as a valuable source of antiviral peptides and highlight the feasibility of using cloud-based bioinformatics pipelines for antiviral peptide discovery. Future studies should focus on validating these results through in vitro antiviral assays and exploring possible peptide optimization strategies for enhanced therapeutic efficacy.

## Figures and Tables

**Table 2:** Protein modeling results on the AlphaFold server.

Peptide ID	Seed	Interface Predicted Template Modeling (ipTM)
A0A8J9SAR9	1743890153	0.52
A0A8J9TDK2	1142432823	0.51
A0A172E6W5	143176737	0.56
A0A8J9SDW0	854280539	0.25
A0A8J9SA87	1972657144	0.5
A0A8J9X3P8	2021447260	0.71

**Table 3:** Swiss-Model modeling results.

Peptide ID	GMQE	QMEANDiscCo	% Identity
A0A8J9SAR9	0.88	0.68 ± 0.11	100
A0A8J9TDK2	0.80	0.45 ± 0.11	100
A0A172E6W5	0.92	0.60 ± 0.11	98.53
A0A8J9SDW0	0.61	0.29 ± 0.11	100
A0A8J9SA87	0.80	0.54 ± 0.12	82.93
A0A8J9X3P8	0.83	0.57 ± 0.11	100

**Table 4:** ClusPro molecular docking results.

Peptide ID	Members	Representative	Weighted Score (kcal/mol)
A0A8J9SAR9	122	Center	−831.2
		Lowest energy	−1214.2
A0A8J9TDK2	132	Center	−867.3
		Lowest energy	−1042.3
A0A172E6W5	90	Center	−799.8
		Lowest energy	−964.1
A0A8J9SDW0	94	Center	−1262.5
		Lowest energy	−1371.5
A0A8J9SA87	103	Center	−805.2
		Lowest energy	−944.3
A0A8J9X3P8	71	Center	−672.6
		Lowest energy	−719.3

**Table 5:** Weak interactions between the tested peptides and human MHC alleles.

Weak Interactions	
Peptide ID	Allele Name
A0A8J9SAR9	HLA-A02:01, HLA-A26:01, HLA-B07:02, HLA-B08-01, HLA-B15:01, HLA-B27:05
A0A8J9TDK2	HLA-24:02, HLA-B08:01, HLA-B15:01, HLA-B58:01
A0A172E6W5	HLA-A02:01, HLA-B07:02, HLA-B08:0
A0A8J9SDW0	HLA-B40:01
A0A8J9SA87	HLA-B07:02, HLA-B40:01
A0A8J9X3P8	HLA-A02;01, HLA-B27:05

**Table 6:** Results obtained from the AVPpred search.

Name	Lenght	Align	Comp	Physico	AVP	Polarity
Reference	9	Non-AVP	49.38%	31.83%	By no method	Hydrophobic = 77% Hydrophilic = 23%
A0A8J9SDW0	62	Non-AVP	18.79%	64.08%	By 1	Hydrophobic = 45% Hydrophilic = 55%
A0A8J9SA87	41	Non-AVP	21.88%	63.38%	By 1	Hydrophobic = 53% Hydrophilic = 47%

## Data Availability

The datasets generated and analyzed during the study are available from the corresponding authors upon reasonable request.
